# Sulforaphane Diminishes the Formation of Mammary Tumors in Rats Exposed to 17β-Estradiol

**DOI:** 10.3390/nu12082282

**Published:** 2020-07-30

**Authors:** Dushani L. Palliyaguru, Li Yang, Dionysios V. Chartoumpekis, Stacy G. Wendell, Marco Fazzari, John J. Skoko, Yong Liao, Steffi Oesterreich, George K. Michalopoulos, Thomas W. Kensler

**Affiliations:** 1 Department of Pharmacology and Chemical Biology, University of Pittsburgh, Pittsburgh, PA 15261, USA; y.lis18@gmail.com (L.Y.); dchartoumpekis@gmail.com (D.V.C.); gstacy@pitt.edu (S.G.W.); maf167@pitt.edu (M.F.); jskoko@pitt.edu (J.J.S.); liaoyong717@outlook.com (Y.L.); oesterreichs@upmc.edu (S.O.); tkensler@fredhutch.org (T.W.K.); 2 Translational Gerontology Branch, National Institute on Aging, Baltimore, MD 21224, USA; 3 Department of Toxic Substances Control, California Environmental Protection Agency, Cypress, CA 90630, USA; 4 Department of Internal Medicine, Division of Endocrinology, University of Patras, 26504 Patras, Greece; 5 Magee Women’s Research Institute, Pittsburgh, PA 15213, USA; 6 UPMC Hillman Cancer Center, Pittsburgh, PA 15232, USA; 7 Department of Pathology, University of Pittsburgh, Pittsburgh, PA 15261, USA; michalopoulosgk@upmc.edu; 8 Translational Research Program, Fred Hutchinson Cancer Research Center, Seattle, WA 98109, USA

**Keywords:** breast cancer, prevention, estrogen, lipid metabolism, sulforaphane

## Abstract

Elevated levels of estrogen are a risk factor for breast cancer. In addition to inducing DNA damage, estrogens can enhance cell proliferation as well as modulate fatty acid metabolism that collectively contributes to mammary tumorigenesis. Sulforaphane (SFN) is an isothiocyanate derived from broccoli that is currently under evaluation in multiple clinical trials for prevention of several diseases, including cancer. Previous studies showed that SFN suppressed DNA damage and lipogenesis pathways. Therefore, we hypothesized that administering SFN to animals that are co-exposed to 17β-estradiol (E2) would prevent mammary tumor formation. In our study, 4–6 week old female August Copenhagen Irish rats were implanted with slow-release E2 pellets (3 mg x 3 times) and gavaged 3x/week with either vehicle or 100 μmol/kg SFN for 56 weeks. SFN-treated rats were protected significantly against mammary tumor formation compared to vehicle controls. Mammary glands of SFN-treated rats showed decreased DNA damage while serum free fatty acids and triglyceride species were 1.5 to 2-fold lower in SFN-treated rats. Further characterization also showed that SFN diminished expression of enzymes involved in mammary gland lipogenesis. This study indicated that SFN protects against breast cancer development through multiple potential mechanisms in a clinically relevant hormonal carcinogenesis model.

## 1. Introduction

Estrogens play a vital role in human health, disease and aging. Even though estrogens are required for the homeostasis of multiple organs, elevated estrogens are associated with a higher risk for certain types of cancers, including cancer of the breast [1,2]. Highly reactive quinones formed during estrogen metabolism have been shown to adduct with DNA leading to DNA damage [3], a process that is tightly regulated by enzymes such as NADP(H): quinone oxidoreductase (NQO1), glutathione-*S*-transferase (GST) and catechol-*O*-methyltransferase (COMT) that catalyze competing pathways that enable clearance of DNA-damaging estrogen metabolites. Although the DNA-damaging effects of estrogen are a likely contributor to breast cancer, there are other prominent mechanisms by which estrogen drives breast cancer emergence [1]. Recently, the role of lipid metabolism within the context of estrogen-mediated cancer has been explored [4]. Specifically, estrogen has been described to play an important role in lipid metabolism, including formation of fatty acids and triglycerides [5].

In addition to the direct genotoxic effects of estrogen itself, the estrogen receptor (ER) is commonly overexpressed in breast cancers and is able to facilitate the activation of signaling cascades that lead to cellular proliferation, survival and angiogenesis [6]. Preventative endocrine therapy with selective estrogen receptor modulators (SERMs) such as tamoxifen has delivered encouraging results in preventing breast cancer, but the utilization of tamoxifen for risk reduction has been limited, in part due to its side effects [7]. Trials with aromatase inhibitors have also shown to be effective for lowering the risk of invasive breast cancer, but bone density loss among other adverse reactions remains a concern [8]. Therefore, exploring alternative breast cancer prevention strategies that are effective, affordable and safe presents a need of the field. Given that the development of new drugs is not only costly but also lengthy, an approach that is based on the repurposing of existing drugs or on making use of food-derived natural products with established safety profiles can potentially foreshorten the process.

Measures of consumption of cruciferous vegetables have shown promising inverse associations with the development of breast cancer in epidemiological studies [9,10]; and notably, with broccoli in observational studies and in biomarker-driven clinical trials [11,12]. The major bioactive component of broccoli, sulforaphane (SFN) has been evaluated in pre-clinical [13,14,15,16,17] and clinical studies [18] as a breast cancer preventive agent. SFN is formed as a result of the hydrolysis of glucoraphanin (the precursor glucosinolate present in broccoli) and is catalyzed by the enzyme myrosinase. SFN is then further metabolized for excretion through the mercapturic acid pathway [19]. Several in vivo studies in rodents have shed light on mechanistic insights of SFN action within the context of breast cancer prevention. However, many of the studies were carried out either in tumor xenograft models or with chemical carcinogens with limited relevance to human exposures. We have shown previously that SFN decreased the formation of depurinating estrogen-DNA adducts in breast epithelial MCF10A cells exposed to metabolites of E2 [20]. SFN partially elicited this response via a nuclear factor erythroid 2 like 2 (Nrf2)-dependent mechanism, driven by altered estrogen metabolism. Additionally, SFN also improves lipid metabolism by enhancing mitochondrial function [21], disturbs maturation of lipid droplets [22] and modulates adipocyte differentiation [23]. Since these multiple modes of action may putatively modulate tumor outcomes, we sought to explore the effectiveness of SFN as a strategy for chemoprevention of estrogen-mediated breast carcinogenesis.

## 2. Materials and Methods

### 2.1. Animal Study

August Copenhagen Irish (ACI) rats harbor chromosomal traits that enhance mammary tumor susceptibility upon exogenous exposure to 17β-estradiol (E2) [24]. Chronic administration of estrogen alone (3–27 mg) or along with progesterone has been shown to induce mammary tumor development in ovary-intact and ovariectomized ACI rats, respectively [25,26].

**Figure 1:**
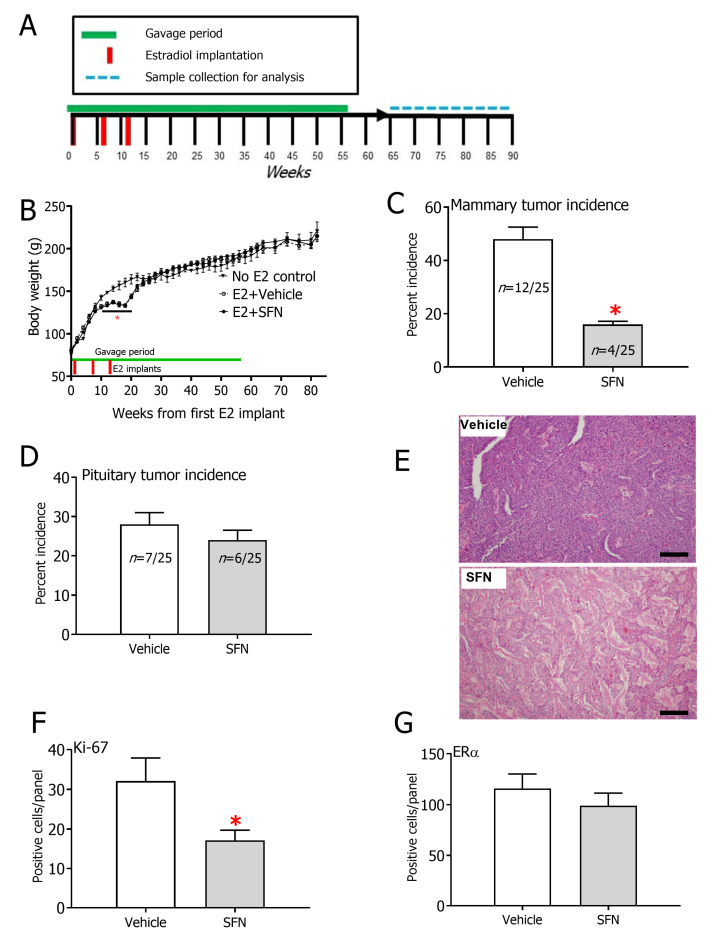
(**A**) Experimental design. (**B**) Trajectories of body weights for rats implanted with E2 pellets that were concurrently gavaged with either vehicle (dimethyl sulfoxide, DMSO) or sulforaphane (SFN). Body weights of age-matched rats that were not implanted or gavaged are also shown (*n* = 6). Incidence of (**C**) mammary tumors and (**D**) pituitary tumors in rats exposed to E2. * *p* < 0.05 (Fisher’s exact test). (**E**) Representative H&E staining images of mammary tumors in vehicle (DMSO) vs. SFN-treated rats. Magnification = 20x, Scale bar = 50 µm. Quantification of (**F**) Ki-67 (**G**) ERα in mammary tumors. (*n* = 3 per group). * *p* < 0.05 (Student’s *t*-test).

Furthermore, mammary tumors arising in ACI rats exposed to E2 are ER+ [27]. Four to six-week-old female ovary-intact ACI rats were obtained from Envigo (Somerset, NJ). After one-week acclimation, rats were randomized and divided equally into two groups: (1) E2+vehicle (dimethyl sulfoxide; DMSO) (*n* = 25) and (2) E2+ sulforaphane (SFN) (*n* = 25). In addition to these animals, six age-matched rats that did not receive E2 were maintained as controls. For the first E2 implantation (week 0), rats were anesthetized with isoflurane, and a single 42-day release 3 mg pellet of E2 (Innovative Research of America, Sarasota, FL, USA) was implanted subcutaneously in the interscapular region. This procedure was repeated two more times at weeks 6 and 12 (Figure 1A). Given the very high dose of E2 used in this model to initiate mammary tumors, it was not necessary to stage the rats according to their normal estrous cycle. The first dose of either DMSO or 100 μmol/kg SFN (LKT Laboratories, St Paul, MN, USA) was administered 24 h before the first E2 implantation. All animals were continued on the DMSO/SFN gavage regimen on Monday, Wednesday and Friday for 56 weeks. Dosing was discontinued at this time as appreciable numbers of DMSO-treated animals began to develop mammary tumors. Animals were provided with ad libitum water and AIN-93 diet (Research Diets, New Brunswick, NJ, USA) throughout the study. Body weights were measured every 10–12 days and food/water consumption were measured at select time points. The primary goal of this experiment was to examine efficacy of SFN against mammary tumor formation, but an effort was made to collect samples for mechanistic insights. Collection of samples for analyses occurred 9–34 weeks after the DMSO/SFN gavage period ended and included both tumor-bearing and non-tumor-bearing rats (Figure 1A). In an antecedent pilot study, ten female ACI rats (6 weeks old) were randomized into 2 groups and were gavaged with either vehicle (corn oil) or 150 µmol/kg SFN for 1 week (3 times/week). All animals were then implanted with a single pellet of 3 mg E2 followed by 6 weeks of the same gavage regimen. At week 7, animals were placed in metabolic cages (Tecniplast USA, West Chester, PA, USA) for urine collection. Urine was collected on ice over a 24 h period and was used for the estrogen-DNA adduct analyses. Serum and tissues (mammary gland and liver) were also collected at this time point and frozen.

### 2.2. Ethics Statement

All animal experiments were conducted according to standards and guidelines established by the Institutional Animal Care and Use Committee (IACUC) of University of Pittsburgh, Pittsburgh, PA, USA who prospectively approved this research (Protocol numbers: 17050661 and 14063807). Animals were anesthetized under isoflurane for pellet implantation and euthanasia.

### 2.3. Blood, Tumors and Organ Collections

Animals were routinely palpated at least once a week for mammary tumors, and when a tumor was observed to reach a size of >0.5 cm in diameter or became necrotic, the animal was euthanized within five days or anytime a rat displayed signs of distress. Blood, tumors and organs were harvested from isoflurane anesthetized animals during sacrifice. Blood was collected via cardiac puncture, let to clot at room temperature and centrifuged for serum collection. Tumors were carefully dissected out in whole, measured for dimensions and weighed. Non-tumor bearing gland contralateral to tumor-bearing gland was collected as normal mammary gland. For non-tumor-bearing rats, inguinal mammary glands were collected. Livers were also collected and weighed. One part of the tumor and organ was snap-frozen in liquid nitrogen and the other part was fixed in buffered 10% formalin overnight.

### 2.4. RNA Isolation from Tissue and Real-Time PCR

Snap-frozen tissue was cut, weighed and homogenized in TRIzol (Invitrogen, Carlsbad, CA, USA). RNA from homogenates was extracted and purified following the manufacturer’s protocol. RNA integrity was confirmed by gel electrophoresis. Quantification of RNA concentration was performed using UV spectrophotometry at 260 nm. Absorbance ratio of 260/280 was utilized to determine the purity of RNA. RNA (1 μg) was used to synthesize cDNA with the qScript system (Quanta Biosciences, Beverly, MA, USA). Primer sequences were obtained from Primer Bank or previous publications (Supplementary data, Table S1). Primer annealing temperatures were determined by semi-quantitative PCR. Real-time PCR was performed on a Bio-Rad My-IQ with SYBR green (Bio-Rad, Hercules, CA, USA) and a StepOne Plus with SYBR green (Applied Biosystems, Foster City, CA, USA). PCR efficiency was determined using a standard curve and melt curve analysis was performed to confirm that each PCR has a single specific product. Pfaffl method was used for quantification of fold changes.

### 2.5. NQO1 Activity

Mammary gland and liver tissue were frozen in liquid nitrogen, stored at −80 °C and were assayed according to protocols described previously [20]. Briefly, tissue was lysed with digitonin with EDTA for 20 min, and NQO1 was assayed by addition of an NADPH-generating system that maintained a constant NADPH concentration −1 mM glucose-6-phosphate, 2 unit/mL glucose-6-phosphate dehydrogenase, 30 µM NADP, as well as 50 µM FAD, 0.24 µM menadione and 0.03 mg/mL MTT. Reaction was stopped after 5 min by adding 50 µM dicumarol and the reduced formazan dye was measured spectrophotometrically at 620 nm.

### 2.6. Immunohistochemistry

Tissue was fixed in buffered 10% formalin overnight; transferred to 70% ethanol and then sectioned, processed and embedded in paraffin. Target antigen retrieval solution at pH 6 (Dako, Carpinteria, CA, USA) was used for antigen retrieval and all slides were treated with 3% H_2_O_2_ in methanol to block endogenous peroxidases. General protein blocking was performed with serum-free blocking solution (Dako, Carpenteria, CA, USA). The slides were stained with antibodies against Ki-67 (Abcam, ab16667), ERα (Santa Cruz, 2Q418) or γ-H2A.X (phospho S139, Abcam, ab26350). The secondary antibodies used were biotinylated goat anti-rabbit IgG (Vector Laboratories, Burlingame, CA, USA; BA-100) for Ki-67, biotinylated goat anti-mouse IgG (Abcam, Cambridge, MA, USA; ab64255) for ERα and γ-H2A.X. All slides were counterstained with hematoxylin and imaged on a Leica CTR5000 microscope (Wetzlar, Germany). Quantification was done on triplicate photomicrographs (depicted as panels in images) by counting positively stained cells by a blinded investigator. Images that are representative of organs from 3 animals are shown in figures. Blinded, non-quantitative histological evaluation of mammary tumors was performed by a pathologist [G.K.M.]

### 2.7. UPLC-MS/MS Analysis of Depurinating DNA Adducts

Extraction of depurinating DNA adducts from urine was modified from previously described procedures [28]. After adjusting the pH to 7, urine was loaded onto phenyl cartridges (Agilent Technologies, Santa Clara, CA, USA) that were preconditioned with methanol and water. Extracts were eluted, lyophilized, re-dissolved in a methanol (water 50:50 mixture containing 0.1% formic acid) and finally subjected to UPLC-MS/MS analysis as described [20]. Analyses were conducted by selected reaction monitoring with a triple stage quadrupole mass spectrometer (TSQ Vantage, Thermo Fisher Scientific, Waltham, MA, USA) by using heated electrospray ionization in positive ion mode.

### 2.8. Sample Preparation and Analysis of Free Fatty Acids and Triglyceride

Serum samples (20 µL) were spiked with 1.27 nmol triheptadecanoin (Nu-Check Prep, Elysian, MN, USA) as internal standard and resuspended into 500 µL phosphate buffer 50 mM pH 7.4. Then, lipids were extracted by Bligh and Dyer procedure, dried and resuspended in ethyl acetate. Mammary gland and liver samples were homogenized using a FastPrep-24 5G homogenizer in 50 mM phosphate buffer pH 7.4 containing BTH (0.16%). An aliquot of the homogenate containing 1 mg/0.5 mL tissue was spiked with 1.27 nmol triheptadecanoin, extracted by Bligh and Dyer procedure, dried and resuspended in ethyl acetate. Targeted fatty acid and triglyceride analysis were performed as previously described [29]. Derivatized fatty acid samples were dried under N_2_ and reconstituted in 1 mL of MeOH, and 5 µL was injected. The other half of the organic phase was dried under N_2_ and reconstituted in 100 µL of chloroform:methanol (2:1), and 5 µL was injected for untargeted lipidomics analysis as described below. All analyses were conducted on a Thermo Fisher Vanquish UHPLC coupled to a Q Exactive hybrid quadrupole-Orbitrap mass spectrometer (Thermo Fisher Scientific, Waltham, MA, USA).

### 2.9. Untargeted Lipidomics

Samples were separated on a Thermo Fisher Accucore C18 column (2.1 X 100 mm, 5 µ pore size) using solvent A (H_2_O:ACN (1:1) with 10 mM ammonium acetate +0.1% formic acid) and solvent B (IPA:ACN (9:1) with 10 mM ammonium acetate +0.1% formic acid) at a flow rate of 0.2 mL/min. The gradient started at 0% B and increased to 50% B from 2–10 min following a second increase to 95% B from 10–47 min. The gradient was held for 4 min at 95% B before increasing to 100% B at 51 min for a 6 min wash. At 57 min, the system was returned to initial conditions to equilibrate before the next injection. Total run time was 60 min. Samples were analyzed using full scan accurate mass at a resolution of 70 K in positive mode and 17.5 K for ddMS^2^. Relative amounts were obtained by taking the area under the peak and normalizing to tissue weight. Thermo Fisher LipidSearch software (Thermo Fisher Scientific, Waltham, MA, USA) was used for identification and relative quantitation of the untargeted analysis.

### 2.10. Statistical Analysis

Values are expressed as mean ± SEM. Unpaired Student’s *t*-test was utilized for statistical comparisons between 2 groups, and one-way analysis of variance (ANOVA) with Tukey’s post-test was utilized for multiple comparisons. For mammary tumor-free survival, non-mammary tumor bearing rats were censored. The Mantel–Cox test was used to compare the Kaplan–Meier mammary tumor-free survival curves between DMSO and SFN-treated rats. For tumor incidence, Fisher’s exact test was used for statistical comparison. *p* < 0.05 was considered to be statistically significant.

## 3. Results

### 3.1. SFN Treatment Leads to Significant Prevention of Mammary Tumor Formation of Rats Exposed to E2

**Figure 2:**
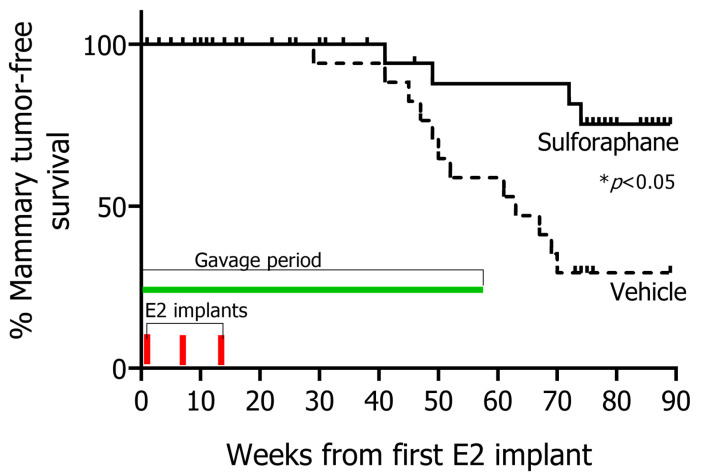
Kaplan–Meier curve for mammary tumor-free survival of rats treated with either vehicle (DMSO) (dotted line) or SFN (solid line). * *p* < 0.05 (Mantel–Cox test). No mammary tumors were observed in age-matched rats without E2 implantation. All non-mammary tumor bearing rats were censored for this analysis.

A marked loss in body weight was observed in the E2-treated rats compared to the untreated controls 10–20 weeks after the first implant, which returned to untreated control animal levels as the third and last exogenously implanted estradiol dissipated (Figure 1B). This transient but significant loss in body weight in the E2-treated rats has also been observed previously [30] in estrogen-treated rats and was linked to the initial post-implantation spike in estrogen levels [31]. There were no differences in body weights amongst treatment groups at the time of cessation of sulforaphane treatment (week 56), indicative of no overt toxicity with this intervention regimen. Interestingly, SFN selectively prevented the formation of mammary tumors (Figure 1C), but not pituitary tumors, which are another common E2-driven tumor type observed in this model; 7 and 6 pituitary tumors were observed in E2+DMSO and E2+SFN groups, respectively (Figure 1D). Pituitary tumors were discovered incidentally during necropsy of rats and had not caused any discomfort or distress to animals. The majority of mammary tumors were detected in the inguinal mammary gland (10/12 in E2+DMSO and 3/4 in E2+SFN). Less frequently, mammary tumors were observed in the thoracic gland (1/12 in E2+DMSO), cervical gland (1/12 in E2+DMSO) and abdominal gland (1/4 in E2+SFN)**.** Overall, there were no differences in the distributions of tumor sites in the mammary tree between the DMSO and SFN-treated rats. No significant differences in tumor volume were seen between DMSO and SFN. No notable differences in tumor multiplicity were observed between DMSO (2 cases of 2 tumors in same rat) and SFN (1 case of 2 tumors in same rat). Pathological evaluation (Figure 1E) suggested high tumor heterogeneity, but no overall pathological differences were observed between vehicle and SFN tumors. Common features observed in many of the tumors included infiltration of inflammatory bodies, mixed differentiation, abscesses arising from tumor death and presence of connective tissue. Mammary tumors arising in these animals were positive for the proliferation marker Ki-67 although SFN-treated rats showed 2-fold lower Ki-67 expression (Figure 1F and Supplemental data Figure S1. To probe differences in expression of ERα and proliferation markers, an immunohistochemical characterization was carried out on selected tumors from both groups. No difference in ERα protein expression was detected between the groups (Figure 1G). Of note, the six age-matched controls that were not exposed to E2 did not develop any mammary tumors or any other tumors that have been described in this model previously. Kaplan–Meier survival curves showed that SFN-treated rats were significantly protected against E2-induced mammary tumor formation compared to their DMSO-treated counterparts (Figure 2).

### 3.2. SFN-Treated Rats Show Altered DNA Damage and Estrogen Metabolism Profiles

**Figure 3:**
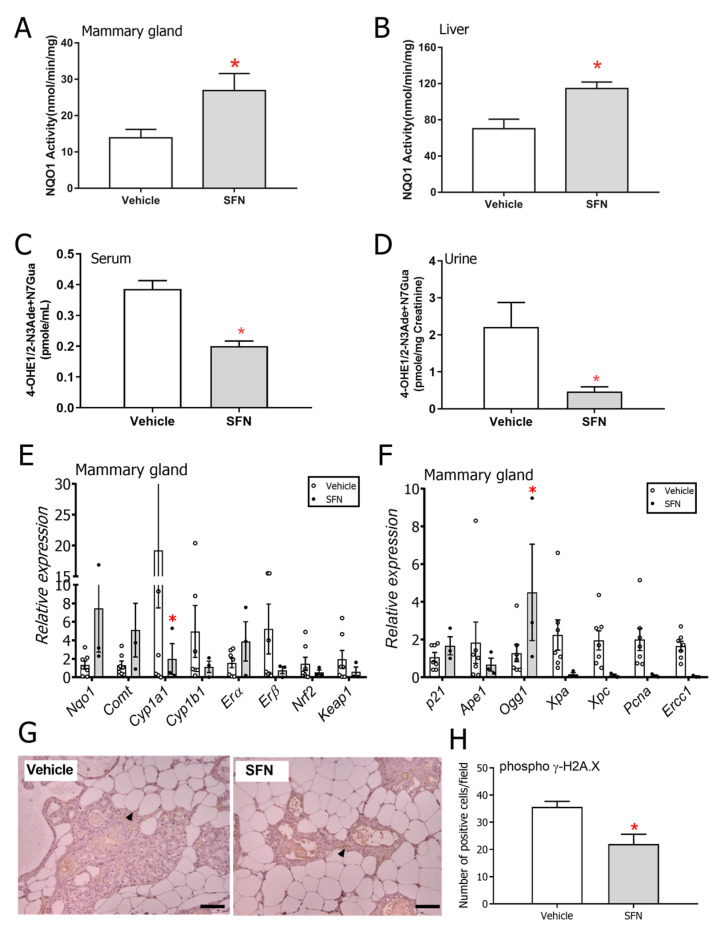
NQO1 activity level in (**A**) mammary gland and (**B**) livers of rats gavaged with vehicle (corn oil) or SFN after 7 weeks of 3 mg E2 implant. Depurinating estrogen-DNA adduct levels in (**C**) serum or (**D**) urine of rats gavaged with vehicle (corn oil) or SFN after 7 weeks of 3 mg E2 implant. (**E**) mRNA expression of NADP(H):quinone oxidoreductase (*Nqo1*), Catechol-o-methyltransferase (*Comt*), Cytochrome P450 1A1 (*Cyp1a1*), Cytochrome P450 1B1 (*Cyp1b1*), Estrogen receptor alpha (*Erα)*, Estrogen receptor beta (*Erβ)*, Nuclear factor erythroid 2–related factor 2 (*Nrf2*), Kelch-like ECH-associated protein 1 (*Keap1*) in the mammary glands of E2-exposed rats by treatment group. *Gapdh* was used as a housekeeping control and expression levels are relative to this. (**F**) mRNA expression of DNA damage repair and response-associated *p21*, *Ape1*, *Ogg1*, *Xpa*, *Xpc, Pcna* and *Ercc1*. *Gapdh* was used as a housekeeping control and expression levels are relative to this. (**G**) Phosphorylated γ-H2A.X immunohistochemistry and (**H**) quantification in the mammary glands of E2-exposed rats treated with either DMSO or SFN. Arrow heads depict representative positively stained cells. Magnification = 20x, Scale bar = 50 µm. All samples used in this analysis were collected after the active DMSO/SFN gavage period and included both mammary tumor-bearing and non-bearing rats. Values are mean ± SEM (*n* = 5 for activity and adduct analysis, *n* = 7 in DMSO group and *n* = 3 in SFN group for transcript analysis, *n* = 3 for immunohistochemistry). * *p* < 0.05 (Student’s *t*-test).

Given that DNA damage is likely a major mechanism by which E2 drives tumor incidence in this model, we examined aspects of this response through a series of assays. Using samples from the pilot experiment where female ACI rats were exposed to 6 weeks of E2, higher NQO1 activity levels were observed in mammary gland (Figure 3A) and liver (Figure 3B) of SFN-treated rats. Elevation of NQO1 by SFN is associated with enhanced detoxication of DNA reactive estrogen metabolites in human mammary epithelial cells in culture [20]. E2 or estrone (E1) can be oxidized to E_1/2_-3,4-quinone, which can bind to DNA to form 4-OHE_1/2_-1-N^3^Adenine or 4-OHE_1/2_-1-N^7^Guanine adducts. NQO1 reduces E_1/2_-3,4-quinones back to catechols. Reduced levels of depurinating estrogen DNA adducts, namely 4-OHE_1/2_-N^3^Adenine and N^7^Guanine adducts were detected in the serum (Figure 3C) and urine (Figure 3D) of the SFN group indicating that SFN indeed modulated the amount of mutagenic DNA adducts that were formed during E2 metabolism in this model [32,33]. In the long-term E2 tumorigenesis experiment, gene expression was quantified in mammary gland samples collected from E2-treated animals that were no longer undergoing treatment with SFN or DMSO (post-week 65 from first E2 implantation). Large inter-individual variability was observed in transcript levels. Transcripts of *Nqo1* and *Comt* (enzymes involved in quinone reduction and clearance of catechol estrogens) trended higher while *Cyp1a1* (*p* < 0.05) and *Cyp1b1* (Cytochrome P450 enzymes involved in the production of hydroxyestrogens [34], the first step in the formation of DNA-damaging metabolites from estrogen) were lower in the mammary glands of E2+SFN rats (Figure 3E). In order to understand whether the mammary glands of SFN-treated rats mount a differential DNA damage response compared to that of DMSO-treated rats, transcripts of representative markers of DNA damage were measured in the mammary glands collected from rats chronically exposed to E2. Expression of *Ogg1* (marker of the base excision repair pathway) was significantly different between groups (Figure 3F). Phosphorylated γH2A.X levels, a biomarker of DNA damage, were ~1.5-fold lower in the SFN-treated mammary gland (Figure 3G,H) suggesting that the overall DNA damage caused by the estrogens was lower in the mammary glands of rats treated with SFN.

### 3.3. SFN Treatment Leads to Decreased Free Fatty Acids and Triglycerides in Serum but not in Mammary Gland and Liver of E2-Treated Rats

**Figure 4:**
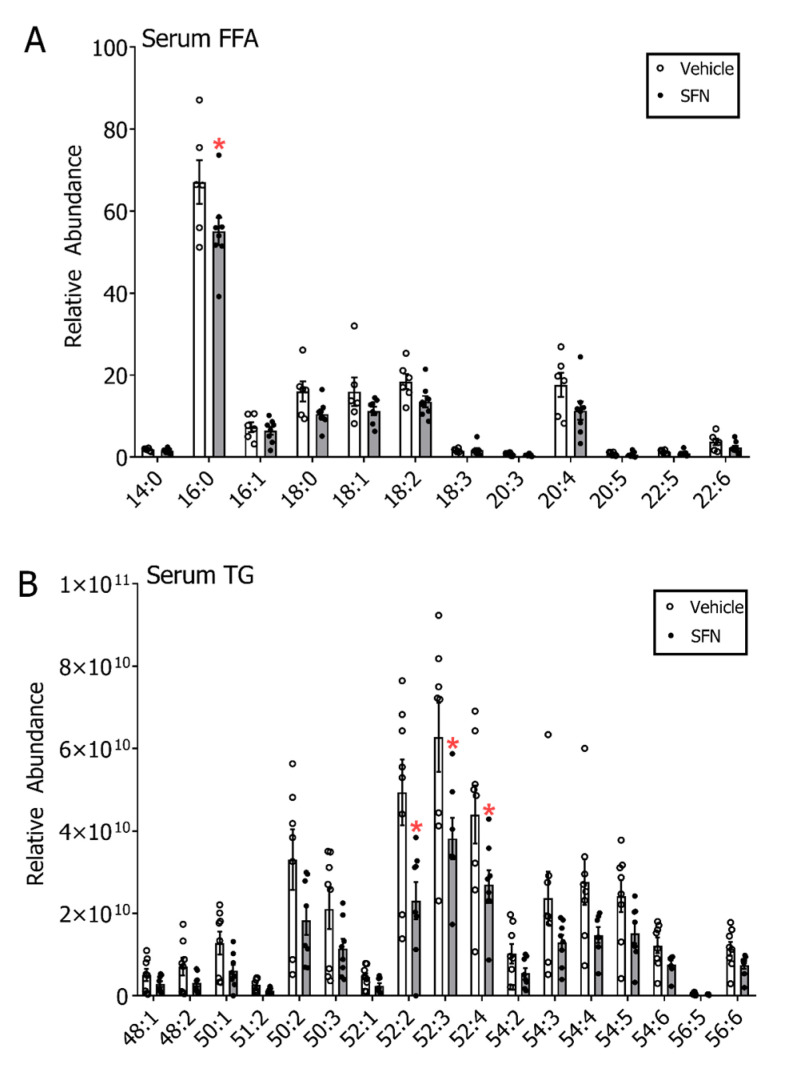
(**A**) Serum free fatty acid (FFA) profile and (**B**) serum triglyceride (TG) profile of rats exposed to E2 by treatment group. All samples included for this analysis were collected after the active DMSO/SFN gavage period and included both mammary tumor-bearing and non-bearing rats. Values are mean ± SEM (*n* = 8 in DMSO group and *n* = 8 in SFN group). * *p* < 0.05 (Student’s *t*-test).

Given recent reports about SFN affecting lipogenesis pathways in cancer [35] and in non-cancer states [36], we evaluated whether SFN could evoke differences in triglycerides and free fatty acid metabolism and thereby affect E2-mediated breast carcinogenesis. Using a UHPLC-HRMS untargeted lipidomics approach we profiled serum lipid classes. Data indicated that there were increases in acyl carnitine, cholesteryl ester, ceramide (Cer), sphingomyelin (SM), phosphatidylcholine (PC), phosphatidylethanolamine (PE), lysophosphatidylcholine (LPC) and lysophosphatidylethanolamine (LPE) sub-classes and decreases in triglycerides (TG) and diglycerides (DG) with SFN treatment (Supplemental data Figure S2). Given the decreases in TG species, we further examined levels of specific free fatty acids (FFA) and TG species. In comparison to the E2+DMSO group, several FFA in the E2+SFN group, such as stearic acid (18:0), oleic acid (18:1), linoleic acid (18:2), dihomo-γ-linolenic acid (20:3), arachidonic acid (20:4) and docosahexaenoic acid (22:6), showed non-statistically significant lower levels while only palmitic acid (16:0) was significantly lower (*p* < 0.05). (Figure 4A). The Scd1 desaturation index was calculated by evaluating the ratios for 16:1/16:0 (0.09, E2+DMSO and 0.12, E2+SFN) and 18:1/18:0 (0.98, E2+DMSO and 1.07, E2+SFN) and showed no difference between the groups. Serum triglycerides were 1.5 to 2-fold lower in SFN compared to DMSO treatments and significant decreases were observed in the most abundant TG, such as 52:2, 52:3 and 52:4 (Figure 4B). Non-statistically significant decreasing trends were similarly observed for all the other TG in SFN treated rats. Inter-individual variability was observed to be notably high in these analyses as well.

**Figure 5:**
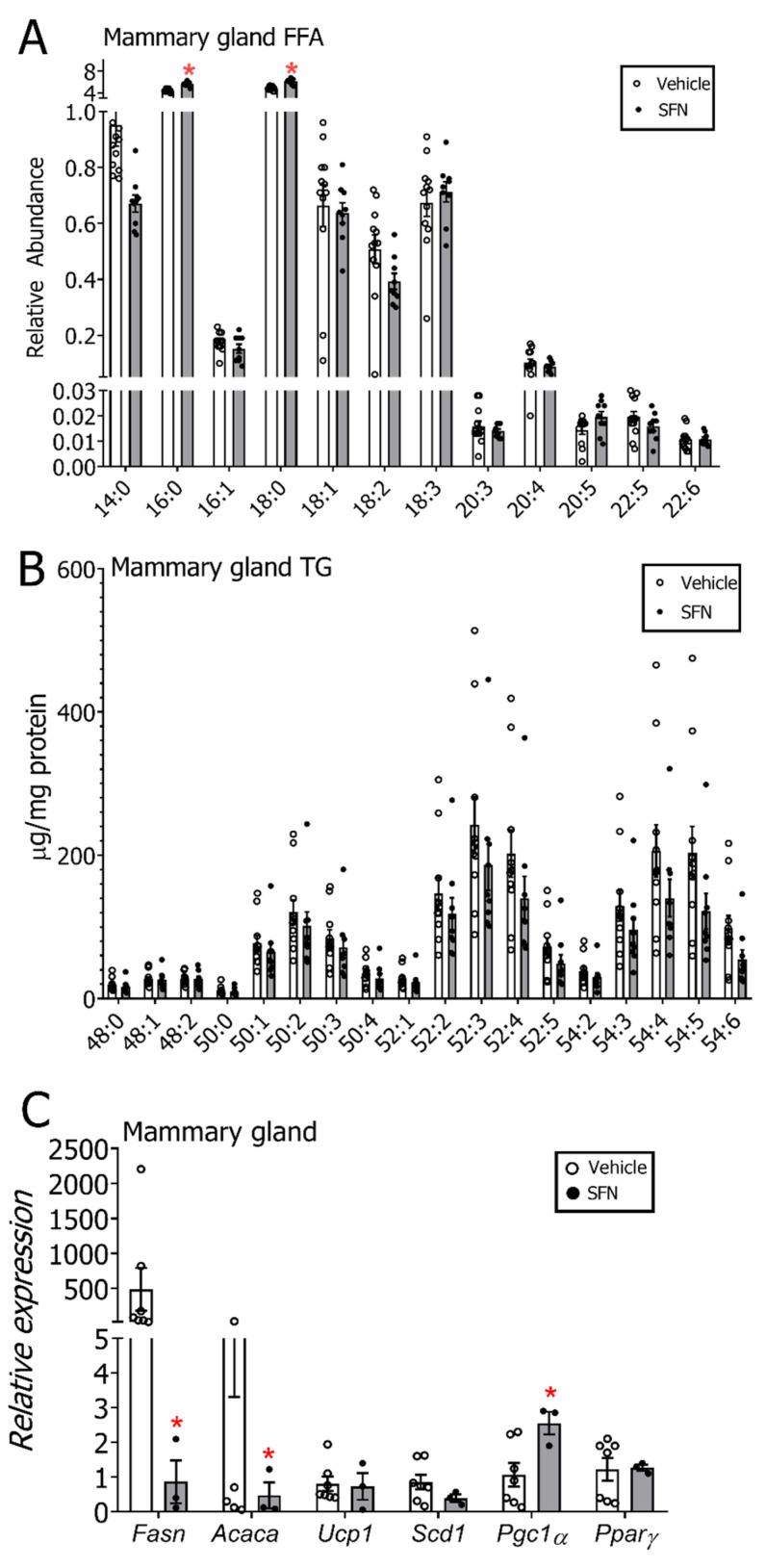
(**A**) Mammary gland free fatty acid (FFA) and (**B**) mammary gland triglyceride (TG) profile by treatment group. (**C**) mRNA expression of Fatty acid synthase (*Fasn*), Acetyl-coA carboxylase 1 (*Acaca*), Uncoupling protein 1 (*Ucp1*), Steayl CoA desaturase 1 (*Scd1)*, Peroxisome proliferator activated receptor gamma coactivator 1 alpha (*Pgc1α)* and Peroxisome Proliferator Activated Receptor Gamma (*Pparγ*) in the mammary glands of E2-exposed rats by treatment group. Expression levels are relative to the housekeeping control *Gapdh*. All samples included for this analysis were collected after the active DMSO/SFN gavage period and included mammary tumor-bearing and non-bearing rats. Values are mean ± SEM (*n* = 12 in DMSO group and *n* = 9 in SFN group for FFA/TG analysis and *n* = 7 in DMSO group and *n* = 3 in SFN group in transcript analysis). * *p* < 0.05 (Student’s *t*-test).

The FFA and TG levels were also analyzed in the mammary gland to evaluate whether the changes observed in serum were compartmental specific. Although some FFA species tended to be lower in the SFN-treated group (14:0, 18:2), in contrast to serum, 16:0 and 18:0 were both slightly enhanced in the mammary glands of the E2+SFN-treated group (Figure 5A). The Scd1 desaturation index was calculated by evaluating the ratios for 16:1/16:0 (24.9, E2+DMSO and 36.9, E2+SFN) and 18:1/18:0 (7.2, E2+DMSO and 9.7, E2+SFN) where the SFN-treated group showed a slightly elevated ratio. Mammary gland TG species displayed an overall decrease in the SFN-treated group, but these changes were not statistically significant (Figure 5B). To understand whether expression levels of enzymes involved in lipogenesis might contribute to changes in mammary gland and circulating lipids, transcript expression profiling of key proteins involved in de novo lipogenesis and fatty acid oxidation was carried out. Results indicated transcript levels of *Fasn* and *Acaca* were significantly downregulated, and *Pgc1α* levels were significantly upregulated in the mammary glands isolated from rats treated with SFN (Figure 5C). Free fatty acids and triglyceride levels in liver samples collected from these rats showed increasing trends overall with a significant increase in 16:0 (*p* < 0.05) (Supplemental data, Figure S3). Gene expression analysis of the liver showed significantly increased *Pgc1α* and *Pparγ* transcripts in the SFN-treated group (Supplemental data, Figure S4).

## 4. Discussion

In the present study, we evaluated the preventative effects of SFN, an agent known to alter estrogen metabolism [20], in the setting of a carcinogenic regimen of E2 and observed that SFN effectively reduced the formation of mammary tumors in E2-exposed ACI rats. While tumor incidence was the primary outcome measured in the study, an exploration of biological mechanisms was conducted which pointed towards enhanced cytoprotection, mitigated DNA damage and suppressed lipogenesis by SFN. Reduced levels of direct (4-OHE_1/2_ adenine and guanine adducts in serum and urine) and indirect (phosphorylated γH2A.X levels in mammary tissue) measures of DNA damage were observed in the SFN treated rats compared to controls.

Although tissue samples were collected 9–34 weeks post final gavage, differences in transcripts of *Cyp1a1, Ogg1*, *Fasn*, *Acaca*, *Pgc1α* (mammary gland) and *17βHsd7*, *Cyp1b1*, *ERβ*, *Nrf2*, *Pgc1α*, *Pparγ* (liver) were observed between DMSO and SFN indicating a non-transient activation of the transcription machinery by SFN. Whether this occurs through an epigenetic imprint or another mechanism is currently unknown. Interestingly, the effect of SFN on lipogenesis appeared to be tissue-specific in this setting–a preliminary finding that warrants further investigation in other models. High inter-individual variability was observed in both groups throughout all assays conducted, which had a major bearing on the final outcomes and their interpretations, and therefore should be carefully considered in future studies.

Obesity is considered to be an important risk factor for breast cancer [37] where in postmenopausal women, it is associated with increased invasive breast cancer risk [38]. However, in premenopausal women, a higher body mass index (BMI) is associated with a protective phenotype against breast cancer [39] indicating a highly complex relationship between obesity, breast cancer risk and hormone status. Ovarian production of estrogens is halted during menopause and the body relies largely on locally-acting estrogens produced by extragonadal organs such as adipose tissue during the post-menopausal phase. The notion that circulating estrogens provide protection against diseases during pre-menopause compared to paracrine estrogen during post-menopause has been considered [40]. While the mechanisms underpinning these associations between hormones, adiposity and cancer risk are not completely understood, reciprocal signaling between cancer-associated adipocytes and cancer cells in the tumor microenvironment seem to facilitate growth and metastasis of tumors [41].

The metabolic component of breast cancer has thereby garnered much attention recently as a way to gain more in-depth understanding of how the phenomena above contribute to hormonally-driven cancer risk [42]. High levels of FFA and TG have been implicated in cellular proliferation and worse cancer-related outcomes [43]. Upregulation of lipogenesis catalyzed by Fasn and Acaca can contribute to this phenotype [44]. Our data from global untargeted serum lipidomics revealed that circulating triglycerides and diglycerides were markedly decreased by SFN while sphingolipids and glycerophospholipids were increased. Recent investigations have shown the important signaling role of sphingolipids in breast cancer where it is surmised that certain sphingolipid species such as ceramides have proapoptotic action, whereas sphingosine exhibits proliferative effects [45]. Increases in acyl carnitine may also indicate increases in fatty acid oxidation [46]. In addition, phospholipids make up a large proportion of cell membranes and therefore are crucial for cellular health and homeostasis. Serum free fatty acids and triglycerides showed an overall decreased trend in the SFN-treated group. The exact role of these different lipid classes and individual species in breast cancer is poorly understood, and it is also noteworthy that serum data represent lipids in circulation and therefore might not reflect tissue-level patterns. In the mammary gland and in the liver, a slightly different trend was observed where palmitate and/or stearate levels tended to be elevated in the SFN group, in contrast to what was seen in serum. This outcome does not reflect the decreased mRNA expression of Fasn and Acaca in the mammary glands of SFN-treated rats. However, the activity of Fasn and Acaca as well as the levels of fatty acids in the mammary glands could have been differentially affected by external stimuli (e.g., hormones or changes in fatty acid flux) [47]. These questions warrant further studies.

The exact mechanism by which these fatty acid species contribute to cancer phenotypes is not well-understood. It is also likely that SFN-mediated lowering of serum FFA and TG was driven by other organs that were not examined during the present study such as adipose tissue or skeletal muscle through possible changes in de novo lipogenesis, lipolysis and fatty acid oxidation. A limitation of the present study was that both tumor-bearing and non-tumor bearing rats were used in both vehicle (DMSO) and SFN groups for lipid profiling to obtain sufficient power for the analysis, which may affect the overall result of serum and tissue free fatty acid and triglyceride concentrations. Cancer can cause changes in whole body metabolism [48], and these can be reflected in lipid species [49]. Future studies examining exactly how such parameters are affected by SFN treatment should also stratify by tumor status. Another major limitation of the study is the fact that samples used for downstream analyses were collected over a period of 25 weeks, which came about because tumor incidence was the major endpoint of this study. Sample collection over such a time period could result in aging-related biological effects. These are important considerations that need to be accounted for in future studies that are designed to answer such specific questions with sufficient power. Aside from the association between lipid profile and tumor status, the interplay between lipid metabolism and DNA damage is another aspect that needs to be considered, especially given that lipid peroxidation and metabolism products can covalently adduct with DNA [50].

## 5. Conclusions

In this study, we showed that SFN significantly inhibited the formation of mammary tumors in ACI rats elicited by a high dose of E2. This is the first such study with long-term administration of SFN in a more physiologically relevant model where cancer is not transplanted or driven by a specific mutation but is the result of chronic estrogen exposure. This protection conferred by SFN was accompanied by an upregulated cytoprotective response yielding decreased DNA damage. Although changes in serum lipid profiles were observed with SFN treatment, these changes were not reflected in the mammary glands despite repressed expression of *Fasn* and *Acaca*. Collectively, these data indicate that SFN might provide a multi-faceted protection against estrogen-driven mammary tumorigenesis.

## Supplementary Materials

The following are available online at https://www.mdpi.com/2072-6643/12/8/2282/s1, Table S1: Realtime PCR primer sequences, Figure S1: Representative images of expression of Ki-67 and ERα in mammary tumors, Figure S2: Untargeted lipidomics on serum collected from E2-implanted rats that were either gavaged with Vehicle (DMSO) or SFN, Figure S3: (A) Free fatty acid (FFA) and (B) triglyceride (TG) quantification of livers from E2-treated rats that were gavaged with Vehicle (DMSO) or SFN, Figure S4: mRNA expression profile in liver tissue between Vehicle (DMSO) and SFN-treated rats.

## References

[1] Yager J.D., Davidson N.E. (2006). Estrogen carcinogenesis in breast cancer. N. Engl. J. Med..

[2] Moore S.C., Matthews C.E., Shu X.O., Yu K., Gail M.H., Xu X., Ji B.T., Chow W.H., Cai Q., Li H. (2016). Endogenous Estrogens, Estrogen Metabolites, and Breast Cancer Risk in Postmenopausal Chinese Women. J. Natl. Cancer Inst..

[3] Santen R.J., Yue W., Wang J.P. (2015). Estrogen metabolites and breast cancer. Steroids.

[4] Hilvo M., Denkert C., Lehtinen L., Muller B., Brockmoller S., Seppanen-Laakso T., Budczies J., Bucher E., Yetukuri L., Castillo S. (2011). Novel theranostic opportunities offered by characterization of altered membrane lipid metabolism in breast cancer progression. Cancer Res..

[5] Palmisano B.T., Zhu L., Eckel R.H., Stafford J.M. (2018). Sex differences in lipid and lipoprotein metabolism. Mol. Metab.

[6] Bjornstrom L., Sjoberg M. (2005). Mechanisms of estrogen receptor signaling: Convergence of genomic and nongenomic actions on target genes. Mol. Endocrinol..

[7] Brown K. (2002). Breast cancer chemoprevention: Risk-benefit effects of the antioestrogen tamoxifen. Expert Opin. Drug Saf..

[8] Visvanathan K., Fabian C.J., Bantug E., Brewster A.M., Davidson N.E., DeCensi A., Floyd J.D., Garber J.E., Hofstatter E.W., Khan S.A. (2019). Use of Endocrine Therapy for Breast Cancer Risk Reduction: ASCO Clinical Practice Guideline Update. J. Clin. Oncol..

[9] Harris H.R., Willett W.C., Vaidya R.L., Michels K.B. (2017). An Adolescent and Early Adulthood Dietary Pattern Associated with Inflammation and the Incidence of Breast Cancer. Cancer Res..

[10] Bosetti C., Filomeno M., Riso P., Polesel J., Levi F., Talamini R., Montella M., Negri E., Franceschi S., La V.C. (2012). Cruciferous vegetables and cancer risk in a network of case-control studies. Ann. Oncol..

[11] Ambrosone C.B., McCann S.E., Freudenheim J.L., Marshall J.R., Zhang Y., Shields P.G. (2004). Breast cancer risk in premenopausal women is inversely associated with consumption of broccoli, a source of isothiocyanates, but is not modified by GST genotype. J. Nutr..

[12] Zhang Z., Atwell L.L., Farris P.E., Ho E., Shannon J. (2016). Associations between cruciferous vegetable intake and selected biomarkers among women scheduled for breast biopsies. Public Health Nutr..

[13] Zhang Y., Kensler T.W., Cho C.G., Posner G.H., Talalay P. (1994). Anticarcinogenic activities of sulforaphane and structurally related synthetic norbornyl isothiocyanates. Proc. Natl. Acad. Sci. USA.

[14] Fahey J.W., Zhang Y., Talalay P. (1997). Broccoli sprouts: An exceptionally rich source of inducers of enzymes that protect against chemical carcinogens. Proc. Natl. Acad. Sci. USA.

[15] Cornblatt B.S., Ye L., Dinkova-Kostova A.T., Erb M., Fahey J.W., Singh N.K., Chen M.S., Stierer T., Garrett-Mayer E. (2007). Preclinical and clinical evaluation of sulforaphane for chemoprevention in the breast. Carcinogenesis.

[16] Li Y., Zhang T., Korkaya H., Liu S., Lee H.F., Newman B., Yu Y., Clouthier S.G., Schwartz S.J., Wicha M.S. (2010). Sulforaphane, a dietary component of broccoli/broccoli sprouts, inhibits breast cancer stem cells. Clin. Cancer Res..

[17] Jackson S.J., Singletary K.W. (2004). Sulforaphane: A naturally occurring mammary carcinoma mitotic inhibitor, which disrupts tubulin polymerization. Carcinogenesis.

[18] Atwell L.L., Zhang Z., Mori M., Farris P., Vetto J.T., Naik A.M., Oh K.Y., Thuillier P., Ho E., Shannon J. (2015). Sulforaphane Bioavailability and Chemopreventive Activity in Women Scheduled for Breast Biopsy. Cancer Prev. Res. (Phila).

[19] Yagishita Y., Fahey J.W., Dinkova-Kostova A.T., Kensler T.W. (2019). Broccoli or Sulforaphane: Is It the Source or Dose That Matters?. Molecules.

[20] Yang L., Zahid M., Liao Y., Rogan E.G., Cavalieri E.L., Davidson N.E., Yager J.D., Visvanathan K., Groopman J.D., Kensler T.W. (2013). Reduced formation of depurinating estrogen-DNA adducts by sulforaphane or KEAP1 disruption in human mammary epithelial MCF-10A cells. Carcinogenesis.

[21] Lei P., Tian S., Teng C., Huang L., Liu X., Wang J., Zhang Y., Li B., Shan Y. (2019). Sulforaphane Improves Lipid Metabolism by Enhancing Mitochondrial Function and Biogenesis In Vivo and In Vitro. Mol. Nutr. Food Res..

[22] Tian S., Lei P., Teng C., Sun Y., Song X., Li B., Shan Y. (2019). Targeting PLIN2/PLIN5-PPARgamma: Sulforaphane Disturbs the Maturation of Lipid Droplets. Mol. Nutr. Food Res..

[23] Valli V., Heilmann K., Danesi F., Bordoni A., Gerhauser C. (2018). Modulation of Adipocyte Differentiation and Proadipogenic Gene Expression by Sulforaphane, Genistein, and Docosahexaenoic Acid as a First Step to Counteract Obesity. Oxid. Med. Cell. Longev..

[24] Gould K.A., Tochacek M., Schaffer B.S., Reindl T.M., Murrin C.R., Lachel C.M., VanderWoude E.A., Pennington K.L., Flood L.A., Bynote K.K. (2004). Genetic determination of susceptibility to estrogen-induced mammary cancer in the ACI rat: Mapping of Emca1 and Emca2 to chromosomes 5 and 18. Genetics.

[25] Shull J.D., Spady T.J., Snyder M.C., Johansson S.L., Pennington K.L. (1997). Ovary-intact, but not ovariectomized female ACI rats treated with 17beta-estradiol rapidly develop mammary carcinoma. Carcinogenesis.

[26] Blank E.W., Wong P.Y., Lakshmanaswamy R., Guzman R., Nandi S. (2008). Both ovarian hormones estrogen and progesterone are necessary for hormonal mammary carcinogenesis in ovariectomized ACI rats. Proc. Natl. Acad. Sci. USA.

[27] Aiyer H.S., Srinivasan C., Gupta R.C. (2008). Dietary berries and ellagic acid diminish estrogen-mediated mammary tumorigenesis in ACI rats. Nutr. Cancer.

[28] Gaikwad N.W., Yang L., Muti P., Meza J.L., Pruthi S., Ingle J.N., Rogan E.G., Cavalieri E.L. (2008). The molecular etiology of breast cancer: Evidence from biomarkers of risk. Int. J. Cancer.

[29] Li X., Frankem A.A. (2011). Improved LC-MS method for the determination of fatty acids in red blood cells by LC-orbitrap MS. Anal. Chem..

[30] Dubuc P.U. (1974). Effects of estradiol implants on body weight regulation in castrated and intact female rats. Endocrinology.

[31] Xu Y., Lopez M. (2018). Central regulation of energy metabolism by estrogens. Mol. Metab..

[32] Li J.J., Li S.A. (1987). Estrogen carcinogenesis in Syrian hamster tissues: Role of metabolism. Fed. Proc..

[33] Liehr J.G., Fang W.F., Sirbasku D.A., Ari-Ulubelen A. (1986). Carcinogenicity of catechol estrogens in Syrian hamsters. J. Steroid Biochem..

[34] Smith S., Sepkovic D., Bradlow H.L., Auborn K.J. (2008). 3,3’-Diindolylmethane and genistein decrease the adverse effects of estrogen in LNCaP and PC-3 prostate cancer cells. J. Nutr..

[35] Singh K.B., Kim S.H., Hahm E.R., Pore S.K., Jacobs B.L., Singh S.V. (2018). Prostate cancer chemoprevention by sulforaphane in a preclinical mouse model is associated with inhibition of fatty acid metabolism. Carcinogenesis.

[36] Kay H.Y., Kim W.D., Hwang S.J., Choi H.S., Gilroy R.K., Wan Y.J., Kim S.G. (2011). Nrf2 inhibits LXRalpha-dependent hepatic lipogenesis by competing with FXR for acetylase binding. Antioxid Redox Signal.

[37] Bianchini F., Kaaks R., Vainio H. (2002). Overweight, obesity, and cancer risk. Lancet Oncol..

[38] Neuhouser M.L., Aragaki A.K., Prentice R.L., Manson J.E., Chlebowski R., Carty C.L., Ochs-Balcom H.M., Thomson C.A., Caan B.J., Tinker L.F. (2015). Overweight, Obesity, and Postmenopausal Invasive Breast Cancer Risk: A Secondary Analysis of the Women’s Health Initiative Randomized Clinical Trials. JAMA Oncol..

[39] Schoemaker M.J., Nichols H.B., Wright L.B., Brook M.N., Jones M.E., O’Brien K.M., Adami H.O., Baglietto L., Bernstein L., Bertrand K.A. (2018). Association of Body Mass Index and Age With Subsequent Breast Cancer Risk in Premenopausal Women. JAMA Oncol..

[40] Simpson E.R. (2003). Sources of estrogen and their importance. J. Steroid Biochem. Mol. Biol..

[41] Park J., Morley T.S., Kim M., Clegg D.J., Scherer P.E. (2014). Obesity and cancer--mechanisms underlying tumour progression and recurrence. Nat. Rev. Endocrinol..

[42] Blucher C., Stadler S.C. (2017). Obesity and Breast Cancer: Current Insights on the Role of Fatty Acids and Lipid Metabolism in Promoting Breast Cancer Growth and Progression. Front. Endocrinol. (Lausanne).

[43] Madak-Erdogan Z., Band S., Zhao Y.C., Smith B.P., Kulkoyluoglu-Cotul E., Zuo Q., Santaliz C.A., Wrobel K., Rossi G., Smith R.L. (2019). Free fatty acids rewire cancer metabolism in obesity-associated breast cancer via estrogen receptor and mTOR signaling. Cancer Res..

[44] Milgraum L.Z., Witters L.A., Pasternack G.R., Kuhajda F.P. (1997). Enzymes of the fatty acid synthesis pathway are highly expressed in in situ breast carcinoma. Clin. Cancer Res..

[45] Sukocheva O., Wadham C. (2014). Role of sphingolipids in oestrogen signalling in breast cancer cells: An update. J. Endocrinol..

[46] Bjorndal B., Alteras E.K., Lindquist C., Svardal A., Skorve J., Berge R.K. (2018). Associations between fatty acid oxidation, hepatic mitochondrial function, and plasma acylcarnitine levels in mice. Nutr. Metab. (Lond.).

[47] Menendez J.A., Vazquez-Martin A., Ortega F.J., Fernandez-Real J.M. (2009). Fatty acid synthase: Association with insulin resistance, type 2 diabetes, and cancer. Clin. Chem..

[48] Lazar M.A., Birnbaum M.J. (2012). De-meaning of metabolism. Science.

[49] Quevedo-Coli S., Crespi C., Benito E., Palou A., Roca P. (1997). Alterations in circulating fatty acids and the compartmentation of selected metabolites in women with breast cancer. Biochem. Mol. Biol. Int..

[50] Chen H., Krishnamachari S., Guo J., Yao L., Murugan P., Weight C.J., Turesky R.J. (2019). Quantitation of Lipid Peroxidation Product DNA Adducts in Human Prostate by Tandem Mass Spectrometry: A Method that Mitigates Artifacts. Chem. Res. Toxicol..

