# Supplementary material for: Sulforaphane Diminishes the Formation of Mammary Tumors in Rats Exposed to 17β-Estradiol
Source: Nutrients. 2020 Jul 30;12(8):2282. doi: 10.3390/nu12082282 (PMC7468750; doi:10.3390/nu12082282)
Supplement: Supplementary file 1 [file nutrients-12-02282-s001.pdf]

Supplemental data (Palliyaguru et al. 2020)

Rat primer ID	Forward sequence	Reverse sequence
Nqo1	GCCATGAAGGAGGCTGCTGT	ATCACCAGGTCTGCAGCTTC
Ucp1	GTGAAGGTCAGAATGCAAGC	AGGGCCCCCTTCATGAGGTC
Fasn	GGATGTCAACAAGCCCAAGT	CAGAGGAGAAGGCCACAAAG
Scd1	TGTTCGTCAGCACCTTCTTG	TCTTGTCGTAGGGGCGATAC
Pgc1α	TGTGCAGCCAAGACTCTGTAT	TATGTTCGCGGGCTCATTGT
Gapdh	GGCACAGTCAAGGCTGAGAATG	ATGGTGGTGAAGACGCCAGTA
Hdac1	TCACCGAATCCGAATGACTCATAA	CTGGGCGAATAGAACGCAAGA
Dnmt1	GCTAAGGACGATGATGAGACG	CTTTTTGGGTGACGGCAACTC
Ape1	TCAGAAAACGTCAGCCAGTG	CGGGAGTTTGTTCTCTGAGC
Ogg1	CTAAGAAGACAGAAGGCTAGGTAG	CTAAGAAGACAGAAGGCTAGGTAG
Nrf2	CACATCCAGACAGACACCAGT	CTACAAATGGGAATGTCTCTGC
Keap1	GGACGGCAACACTGATTC	TCGTCTCGATCTGGCTCATA
Pparγ	CAGCCACCATCAACGCAAGT	TTACAGCTCAGTTGAACGCCTTTTG
Pcna	ACTTGGAATCCCAGAACAGG	CACAGCATCTCCAATATGGC
Ercc1	AAGGCGTATGAGCAGAAGC	TCCAAATGTAGTGAGGAGGGT
Xpc	CAGCCTTTGCCCACCTCCA	TCCACGACAATACCCAAGGAC
Xpa	AAGAAGAACCCTCGCCATTCA	TTCAAGAGCCCGCTTTACAAC

Supplemental Table S1. Realtime PCR primer sequences

CONFIDENTIAL

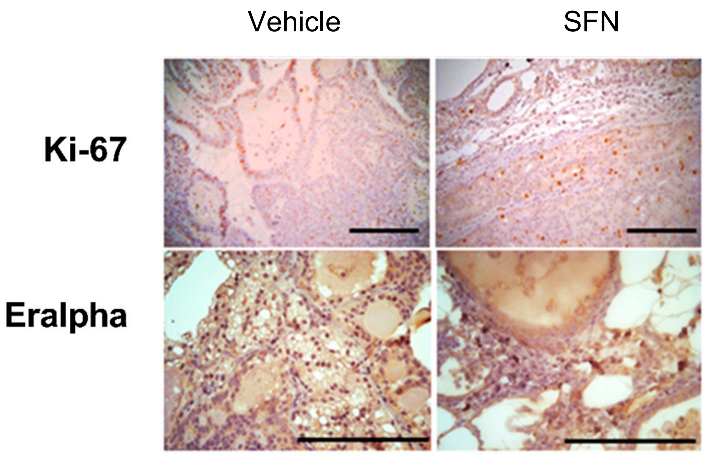

Supplemental Figure S1. Representative images of expression of Ki-67 and ERα in mammary tumors. Images present n=3 per group. Scale bar = 50 µm.

CONFIDENTIAL

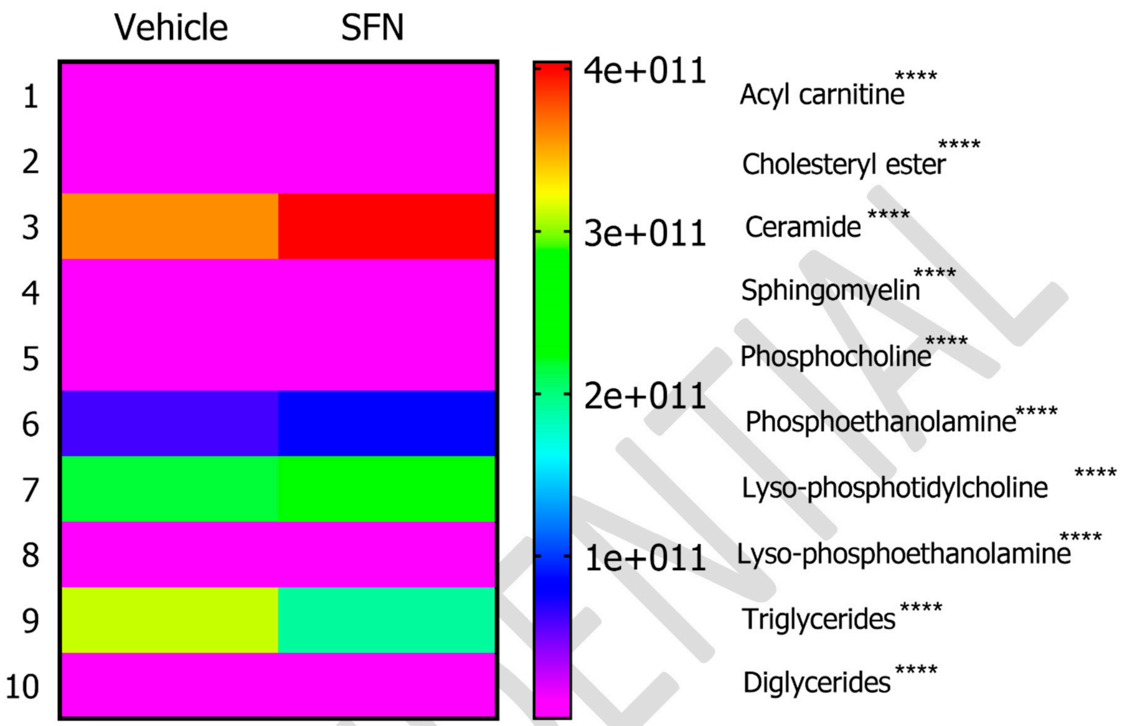

Supplemental Figure S2. Untargeted lipidomics on serum collected from E2-implanted rats that were either gavaged with Vehicle (DMSO) or SFN. All samples included for this analysis were collected after the active DMSO/SFN gavage period and includes both mammary tumor-bearing and non-bearing rats. Values are mean ± SEM (n=8 per group). ****p<0.0001 (Student's t-test) compared to DMSO.

CONFIDENTIAL

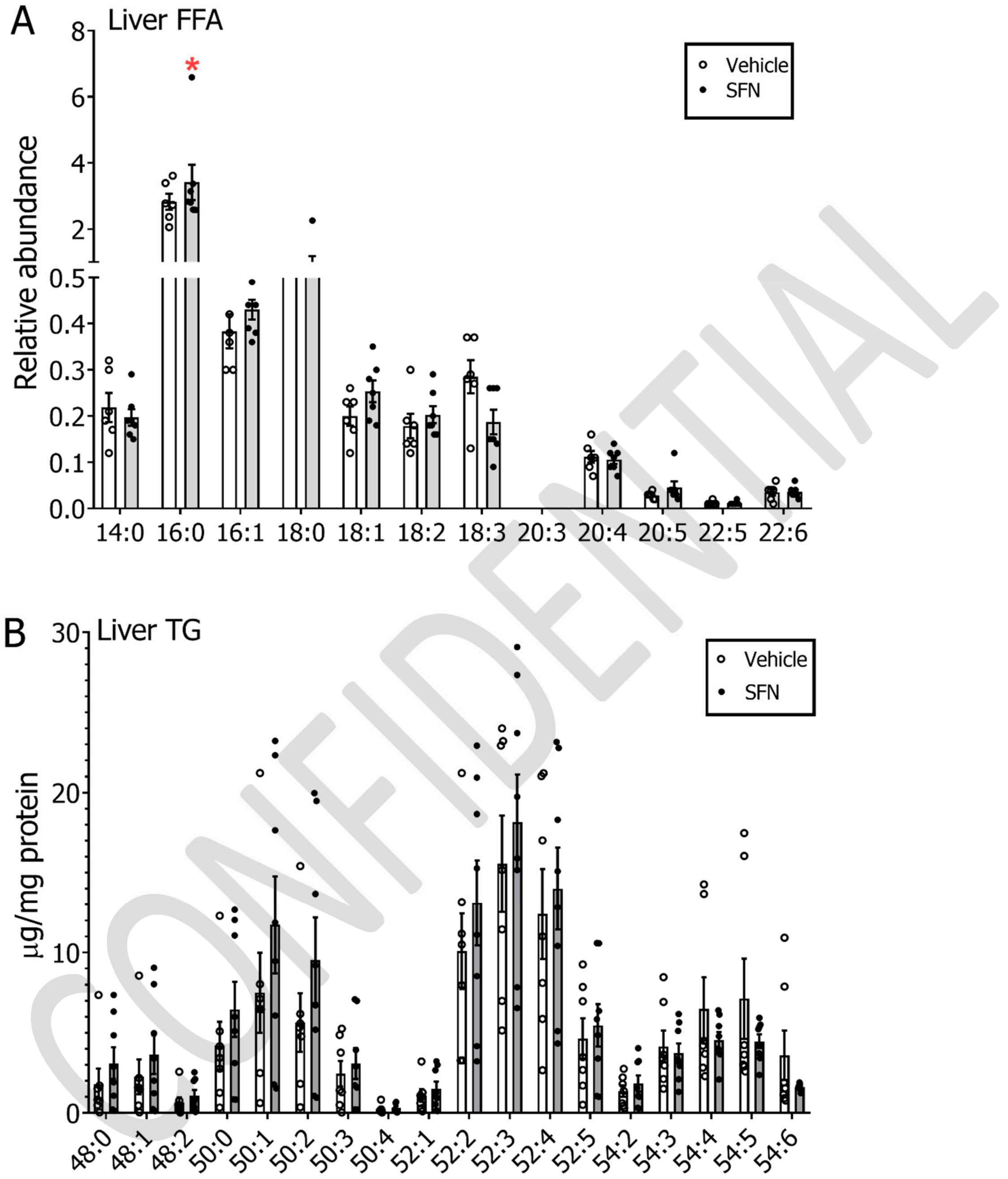

Supplemental Figure S3. A) Free fatty acid (FFA) and **B)** triglyceride (TG) quantification of livers from E2-treated rats that were gavaged with Vehicle (DMSO) or SFN. All samples included for this analysis were collected after the active DMSO/SFN gavage period and includes both mammary tumor-bearing and non-bearing rats. Values are mean ± SEM (n=12 in DMSO group and n=9 in SFN group). *p<0.05 (Student's t-test).

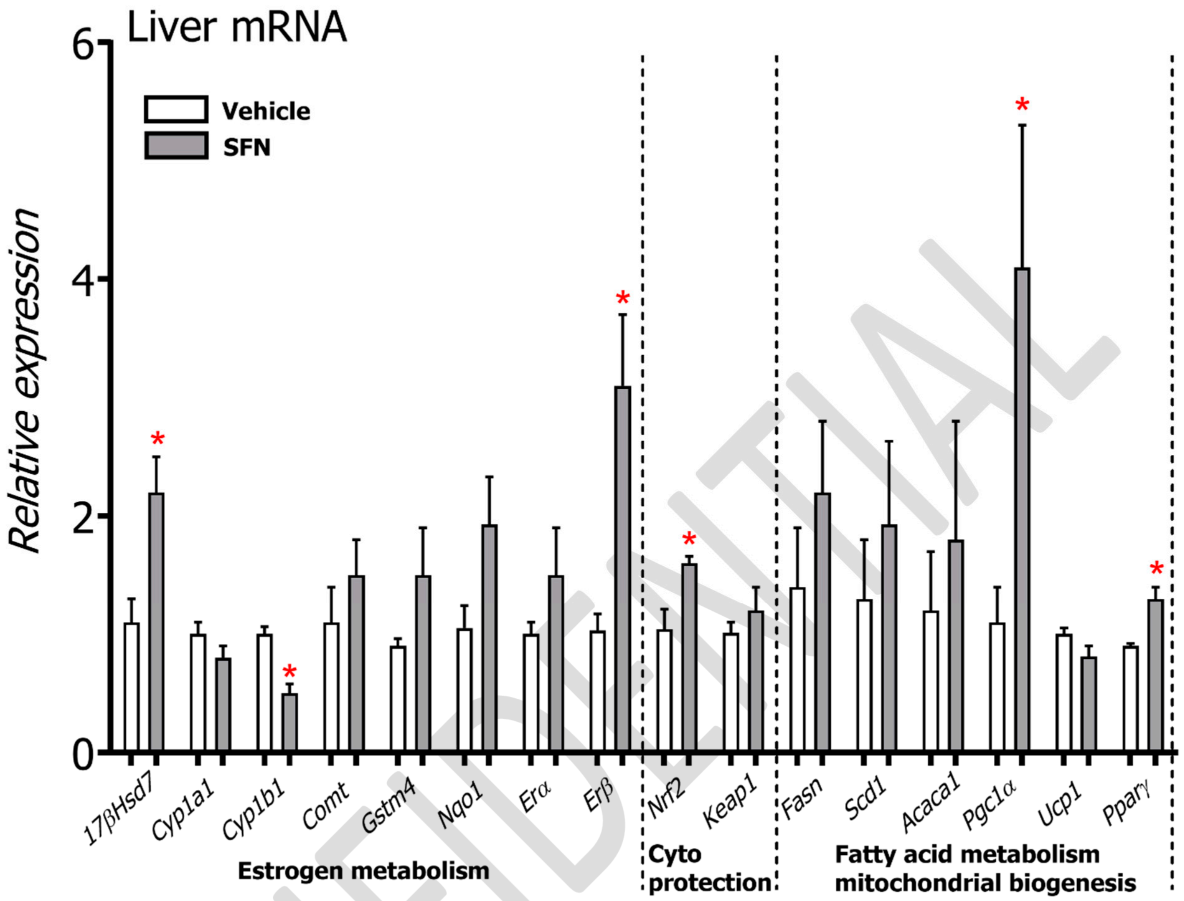

Supplemental Figure S4. mRNA expression profile in liver tissue between Vehicle (DMSO) and SFN-treated rats. All samples included for this analysis were collected after the active DMSO/SFN gavage period. *Gapdh* used as housekeeping control. Values are mean ± SEM (n=7 in DMSO group and n=3 in SFN group). *p<0.05 (Student's t-test).

CONFIDENTIAL
